# Embedding Equity, Transparency, and Impact Through Patient and Public Involvement: Insights From the HANDCLAP Study

**DOI:** 10.1111/hex.70790

**Published:** 2026-08-04

**Authors:** Madhavi Seshu, Judith Humphreys, Gillian McCarthy, Alison Mcloughlin, Sondos Albadri

**Affiliations:** ^1^ Alder Hey Children's Hospital NHS Trust Liverpool Merseyside UK; ^2^ University of Liverpool, School of Dentistry and Alder Hey Children's Hospital NHS Trust Liverpool Merseyside UK; ^3^ Cleft Lip and Palate Association (now known as Cleft Lip and Palate Action) London UK; ^4^ NIHR, ARC North West Coast Preston Lancashire UK; ^5^ Current affiliation: East Lancashire Hospitals NHS Trust Blackburn Lancashire UK; ^6^ University of Liverpool, School of Dentistry and Alder Children's Hospital NHS Trust Liverpool Merseyside UK

**Keywords:** cleft lip and palate, inclusive research, oral health inequalities, patient and public involvement, PPIE frameworks

## Abstract

**Introduction:**

Patient and public involvement and engagement (PPIE) in research refers to studies conducted with or by members of the public, rather than for them. The Health Inequalities Assessment Toolkit (HIAT) aims to integrate an intersectional equity perspective into research and considers how people with real‐life experience can contribute to this process. The Guidance for Reporting Involvement of Patients and the Public (GRIPP2) is an international tool that standardises the reporting of PPIE in healthcare projects. There is growing interest in assessing the impact of public involvement (PiiAF) on both researchers and public contributors. The objectives of PPIE for the HANDCLAP study (Health inequalities in access to Dental care for children with Cleft Lip And Palate) was to ensure that parent and public voices were included at all stages of the qualitative research process, from study design through dissemination.

**Methods:**

PPIE in this study was guided by the HIAT, GRIPP2 and PiiAF frameworks. HIAT assessments were conducted at three points during the project to ensure that equity was sustained. GRIPP2 was applied to report the aims, methods, outcomes, conclusions and reflections of PPIE. The Public Involvement Impact Assessment Framework (PiiAF) was used to assess the impact of PPIE.

**Results:**

Parents and public contributors participated in the following stages: proposal development, review of information sheet and consent forms, feedback on translated Welsh documents, PPIE discussion group, development of the visual topic guide and summary, feedback on qualitative interview questions and dissemination of findings.

**Conclusions:**

This article shows how structured PPIE was used to improve research design, accessibility and relevance of the HANDCLAP study. Using established frameworks supported the consideration of equity, transparency and impact. Embedding PPIE at all stages is important in research addressing health inequalities.

**Patient or Public Contribution:**

Parents with lived experience were involved at all stages of the study. A diverse group of Parent helped the research team by giving feedback on study documents (such as the information sheet and consent form), helping to design better interview questions, and explaining why certain families find it difficult to access dental care. They shared their experiences of accessing dental care, which helped identify barriers, particularly for underserved groups, and informed changes to the study design, recruitment, and data collection for HANDCLAP study. Parent involvement also made it possible to develop more accessible and diverse outputs, including a visual summary of PPIE activities (Figure 2), animation video, and plain‐language materials. These diverse outputs reflected parents' preferences for how findings should be shared. Parents were also involved in dissemination, including attending a national webinar.

**Abbreviations**
ARC NW: Applied Research Collaboration, North West Coast
CLAPA: Cleft lip and Palate Association (now Cleft Lip and Palate Action)
CLEAR: Cleft Lip Education with Augmented Reality
GRIPP: Guidance for Reporting Involvement of Patients and the Public
GRIPP2‐ SF: Guidance for Reporting Involvement of Patients and the Public 2 ‐ Short Form
HANDCLAP: Health inequalities in access to Dental care for children with Cleft Lip and Palate
HIAT: Health Inequalities Assessment Toolkit
MDT: Multidisciplinary team
NIHR: National Institute for Health and Care Research
PiiAF: Public involvement impact Assessment Framework
PPIE: Patient and Public Involvement and Engagement
UK: United Kingdom

## 1 Introduction

The primary aim of the publicly funded healthcare system is to improve health and well‐being at both the individual and population levels within finite resources. Consequently, the development and implementation of healthcare policies based on robust evidence are essential [1]. However, the idea that the translation of research evidence into clinical practice is a linear sequence of steps oversimplifies the process [2].

Patient and public involvement and engagement (PPIE) is now widely recognised as an important component of health research, especially where care is long‐term and delivered through multidisciplinary teams. PPIE refers to research undertaken *with* or *by* patients, carers, and members of the public, rather than *to* or *about* them. This approach recognises that lived experience provides important knowledge that complements clinical and academic expertise. Increasing attention has also been given to the importance of equity and inclusion within PPIE. There are concerns that PPIE activities may engage a narrow group of contributors, thereby failing to reflect the diversity of those affected by health conditions and potentially reinforcing existing inequalities [3].

These considerations are especially relevant in cleft and craniofacial care, where disparities related to socioeconomic circumstances, ethnicity, geography, and access to specialist services have been identified. Individuals and families often remain connected to services from infancy into adulthood, attending multidisciplinary team (MDT) clinics and navigating complex treatment pathways. These experiences place patients and families in a strong position to comment on what matters in research, which outcomes are meaningful, and how research processes are experienced in practice [4].

PPIE in research includes working with the public to decide what should be investigated, offering advice as members of a group, and commenting on and developing research materials. A member of the PPIE group within a cleft lip and palate research programme reported that being part of an inclusive group to make a change to cleft research and help more people understand what life is like with a cleft both gives purpose to a patient journey and makes research more accurate [5]. According to a 2023 survey of members of the Cleft Lip and Palate Association (CLAPA, a national cleft charity), 94% agreed that cleft researchers should seek feedback about their research projects from people affected by cleft conditions [6].

The reasons for involving patients and their families in research are well established, including an ethical argument grounded in respect and shared decision‐making, a democratic consideration linked to accountability for publicly funded work, and a practical case. Evidence suggests that PPIE can strengthen research by helping researchers focus on relevant questions, refine study materials, and improve recruitment and retention [7]. In cleft and craniofacial research, this practical contribution is particularly important. Surgical outcomes alone do not capture the full impact of cleft care; speech, appearance, oral health, psychosocial wellbeing, and participation in everyday life are equally central to how patients and families judge success.

The Cleft Clinic Passport, developed with patient and public input, aimed to improve children's understanding of MDT clinics and their experience of attending services [8]. More recently, the Cleft Lip Education with Augmented Reality (CLEAR) project placed parents and patients at the centre of developing an educational resource, using participatory methods to ensure that the final product reflected family priorities and was accessible in real‐world settings [9]. These studies show how involvement can lead to practical outputs that fit naturally within cleft care pathways. Bates et al. [10] have called for greater recognition of individuals with lived experience as ‘experts by experience’ in craniofacial research, and their perspective highlights the value of such expertise.

Despite increasing expectations from funders and journals, PPIE is often reported in limited detail. While many studies state that patients or parents were involved, they provide little information regarding who was involved, what they were asked to do, or how their input influenced decisions, making it difficult for readers to judge the quality of involvement or learn from previous work. There remains limited evidence on how PPIE is applied in practice using structured frameworks, and how it influences research design and outcomes in studies addressing health inequalities.

Concerns about inconsistent reporting led to the development of the Guidance for Reporting Involvement of Patients and the Public (GRIPP) checklist, later revised to the GRIPP2. The GRIPP2 was developed to support clearer and more consistent reporting of PPIE. Two versions are available: a long form for studies where PPIE is a central focus, and a short form for studies where involvement is a secondary component [11].

There is also growing interest in understanding the impact of PPIE, rather than simply documenting its presence. Impact may be seen in changes to study design, outcome selection, or research processes, as well as in less visible shifts in how researchers and contributors view their roles [7]. The Public Involvement Impact Assessment Framework (PiiAF) supports structured reflection on how PPIE may influence research by considering the context of the study, how involvement is carried out, and its potential impact [12].

Health inequalities are vast and avoidable, representing unjust differences in health outcomes. These inequalities arise from intersecting forms of disadvantages, incapability, and discrimination across socioeconomic classes, gender, ethnicity, sexuality, age and disability. The Health Inequalities Assessment Tool (HIAT) offers a structured way to consider equity throughout a research project. By prompting reflection at different stages of the study, it supports more deliberate consideration of who is involved, who may be missing, and how involvement practices might influence inequalities [6, 13].

Together these PPIE frameworks support more inclusive and equity‐focused research, particularly in complex settings such as cleft care.

Within this context, the HANDCLAP study was conducted in North West England and North Wales to investigate social and health inequalities faced by children with cleft lip and palate regarding access to dental care. The study used qualitative methods to explore access to dental care for children with cleft lip and palate and understand how this access is affected by social and health inequalities. This article aims to describe the PPIE activities undertaken, evaluate the impact on the qualitative research process, and focus on equity, transparency, and impact using established PPIE frameworks.

## 2 Methods

This article reports on Patient and Public Involvement and Engagement (PPIE) activities undertaken to inform the qualitative study (HANDCLAP). We evaluated the impact, equity, and transparency of PPIE activities using established frameworks (HIAT, GRIPP2 and PiiAF). HIAT was used to consider equity at different stages of the study, GRIPP2 was used to guide and report PPIE activities, and PiiAF was used to assess the impact of PPIE.

### 2.1 Ensuring Transparent Reporting, Assessing Inequalities and Measuring Impact

The HIAT [13] supported the integration of an intersectional equity lens into our research and highlighted how people with lived experience, can contribute to this process. The HIAT assessments were conducted at three time points during this study: at the start of the project (prior to proposal submission), after the PPIE group discussion (prior to ethical approval), and after the completion of interviews and data analysis. A discussion with a senior health equity researcher enhanced understanding of the concept of an ‘equity lens.’

Throughout the project, the HIAT was used as a practical, note‐based aid to support decision‐making and record equity‐related issues as they arose. The HIAT was not used as a checklist, but as a way of structuring brief reflections during key stages of the study, including PPIE activities, data collection, and planning for dissemination. The notes focused on who was taking part, whose views were shaping decisions, and whether any barriers to involvement were becoming apparent. When potential inequalities were identified, this led to adjustments in the research process, such as changing how information was shared, allowing more time for engagement, or adapting dissemination plans to improve accessibility.

The GRIPP2 short form (GRIPP2‐SF) was used for reporting public involvement in the study, covering aims, methods, results, outcomes, and critical perspectives [14, 15, 16]. The GRIPP2‐SF was applied at two time points in this study – during the project and after completion – for reflections and critical perspectives.

The GRIPP2 was used as a simple way to keep track of PPIE during the project as the work progressed. The team noted why PPIE was included, how people were involved, and whether their input led to any changes. These notes helped make sure involvement was considered throughout the project and provided a clear record to draw on when reporting PPIE at the end of the study. Reflections was an important part of the GRIPP2 because they showed what PPIE looked like in practice; they helped explain what worked, what was difficult, and how involvement influenced decisions during the study.

An impact assessment was conducted both during and after the study using the PiiAF [12]. The PiiAF is grounded in values of respect, inclusion, and partnership, and encouraged our research team to think carefully about why involvement is being undertaken and what it is intended to achieve. Importantly, the framework acknowledges that the impact of PPIE may be both short‐ and long‐term. The PiiAF prompted consideration of practical issues, such as time, resources, skills, and organisational support, which influenced how involvement works in practice. The short‐term impacts were immediate and visible, such as changes to study materials or procedures, while others emerged over time, including shifts in researcher thinking and influence on research culture and priorities. The impact assessment question was as follows: Does involving parents from diverse backgrounds in a PPIE discussion group lead to more credible, culturally sensitive, and relevant findings for a multi‐cultural society?

The HANDCLAP study was undertaken as part of the National Institute for Health and Care Research, Applied Research Collaboration, North West Coast (NIHR ARC NW) Internship. The qualitative study findings, intersectionality, and subsequent real‐world implementation are being prepared for submission as a seperate publication, titled From Barriers to Equity: Understanding Dental Care Access for Children with Cleft Lip and Palate (HANDCLAP Study) CLAPA (Cleft Lip and Palate association) is a UK‐based charity. In this article, ‘CLAPA team members’ refers to staff involved in facilitating PPIE activities, and the ‘CLAPA mediated discussion group’ refers to the PPIE sessions organised and facilitated by the CLAPA team. The PPIE group included the following:

1. A group of three parents with lived experience of cleft lip and palate, recruited by clinical professionals at the beginning of the study after healthcare appointments.
2. A public contributor (school headteacher) and a paediatrician to assess the consent forms, information sheets, and study proposal; and a parent to check the Welsh translation.
3. A diverse group of four parents with lived experience was recruited nationally, and three were recruited locally in Liverpool to be part of a CLAPA‐mediated discussion group of seven parents.
4. CLAPA team members with prior experience in impact reporting and communications.
5. A graphic artist to develop a visual topic guide and summary.
6. Four parents were involved for feedback on the animation storyboard.
7. Parents from the ‘CLAPA mediated discussion group’ were invited to attend a national webinar for dissemination of findings of the study.

### 2.2 Establishing a Diverse PPIE Group

Recruiting PPIE members from diverse socioeconomic and cultural backgrounds was achieved by directly engaging with parents locally and explaining the need for PPIE. The recruitment of participants from underserved groups was facilitated by explaining clearly, and in simple language, how the results of the study directly affected them.

Additionally, CLAPA advertised the PPIE opportunity on its website for a national perspective. To ensure inclusivity, the advertisement was written in simple language, without any conditions for involvement and using the lay title of the study. The PPIE lead for this study held multiple discussions with the CLAPA to encourage diversity in participation.

PPIE discussion sessions lasted between 30 min and 1.5 h, depending on the input required. Some parents were unable to participate due to time constraints. Most PPIE volunteers were female, except for one, and recruiting male participants for discussion groups proved to be difficult.

Language barriers also limited participation, as some believed they would not be able to follow the discussion in the PPIE group. Including interpreters was not feasible in online sessions; therefore, participants whose first language was not English, but who could communicate effectively in English were recruited.

### 2.3 Conducting the PPIE Sessions

CLAPA team members suggested using a vignette to guide discussion with parents, provide context, clarify individual judgements, and act as an ‘icebreaker’ at the start of the session. The story was hypothetical but intended to be relatable to parents' real‐life experiences.

The vignette used was: ‘Sam visited the cleft clinic at the hospital with his dad when he was 5 years old. The specialist dentist wrote to Sam's regular dentist recommending fillings and fluoride varnish. When Sam turned 7 years old, he returned to the clinic for assessment, but unfortunately, no dental treatment had been carried out, and the doctors had to delay his bone graft operation. Sam's mouth was not healthy enough for surgery.’

PPIE discussion was conducted after working hours via an online video platform. CLAPA team members obtained consent from each parent by email prior to the sessions. Parents were informed that a graphic artist would participate to produce visual minutes for the topic guide. The principal investigator of this study attended the first part of the discussion as an observer. The second part involved a more in‐depth discussion of the issues raised in the first part to explore topics such as parents' priorities.

Feedback on the animation storyboard was done after clinical appointments so that parents could visualise the images and provide instinctive feedback.

### 2.4 Funding of PPIE

PPIE was funded by Alder Hey Children's Charity, and remuneration was provided in accordance with the NIHR guidelines for the time and effort contributed by the parents/guardians.

## 3 Results

### 3.1 Understanding the Dimensions of Oral Health Inequalities

Following PPIE discussion, parents identified socioeconomic factors—including deprivation, culture, language, education, homelessness, availability of local dental care, and disability—as relevant to inequalities in access to dental care. Additionally, some of these factors may intersect. For example, a parent could face both language and disability barriers, which together impede access to dental care. Parents suggested that many of these inequalities may stem from the unequal distribution of resources. In response to these discussions, the research team incorporated data collection during recruitment, to monitor participant diversity and inform decisions to support equity and transparency throughout the project.

These discussions also highlighted structural factors such as education and socioeconomic background as important influences on access to care. Based on these insights, open‐ended interview questions were developed to explore parents' experiences of accessing dental care. The involvement of research team members from diverse cultural backgrounds helped understanding of parents' hesitation to participate in research, particularly regarding cultural practices and language barriers.

### 3.2 Understanding Barriers to Access Dental Care Through Lived Experiences

Parents interpreted the vignette differently based on their individual circumstances and previous experiences. For example, a parent whose child was treated in the community dental service reported a different perspective from that of a parent who had relocated to the United Kingdom and was unable to access primary dental care. Parents discussed their experiences of accessing dental care for their child outside the hospital, highlighting how these experiences shaped their understanding of barriers to care. Based on these insights, the study design incorporated open‐ended questions to capture diverse lived experiences of accessing dental services, including variations in access to community and primary dental care. The animation storyboard prompted diverse reactions based on experiences and circumstances.

### 3.3 Developing the Research Question

The research question for the HANDCLAP study was developed in collaboration with PPIE contributors and peer‐reviewed by researchers and clinicians before the start of the project. PPIE contributors helped to simplify the title of the HANDCLAP study to: What are the barriers to access dental care for children with cleft lip and palate after an assessment by a specialist in children's dentistry?

### 3.4 Outcomes of the PPIE Discussion

The PPIE discussion was guided by the PiiAF, with key points recorded for impact assessment. Ground rules were established by CLAPA team and principal investigator to ensure that all parents were able to express their views. No apparent tension was observed between research team members regarding their values; it was acknowledged that disagreements could arise and that diverse viewpoints could provide valuable insights. Parents highlighted that barriers to dental care may differ depending on the population considered, emphasising the importance of involving underserved groups. In response to PPIE discussions, the HANDCLAP study was designed to explore the barriers to accessing dental care outside hospital settings among children with cleft conditions. Parents emphasised the importance of accessibility and inclusivity, and as a result, research documents were revised to use simpler and clearer language. Innovations such as a language‐adjustment chatbot and immersive reading technology were introduced to support accessibility. Cultural and socioeconomic background were considered while designing open‐ended interview questions. Questions exploring parent‐related factors contributing to barriers to accessing dental care were designed in collaboration with CLAPA team members and parents to ensure that they were appropriate and sensitive.

**Figure 1:**
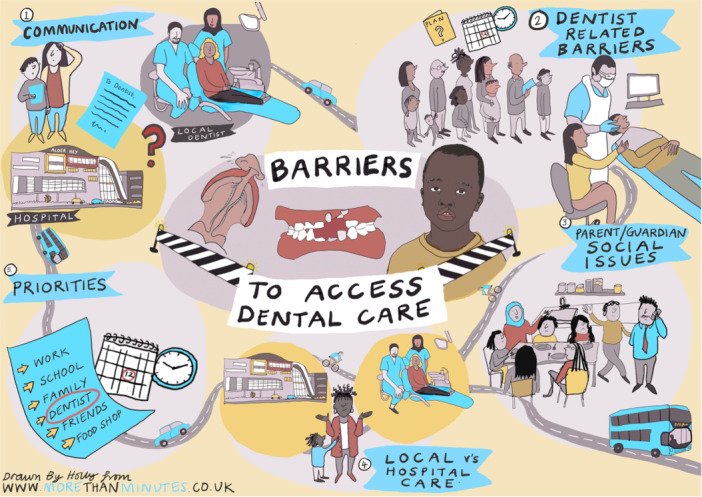
Visual topic guide.

Practical challenges included difficulties scheduling a PPIE group session, time constraints, language barriers, and the reluctance of some parents to speak in groups. These were addressed through careful planning, managing technical issues with virtual platforms, and ensuring diversity in the PPIE group. The PPIE discussion was illustrated by a graphic artist to create a visual topic guide for HANDCLAP qualitative study, which clarified key themes in an accessible format. (Figure 1). Parents in the PPIE group strongly recommended offering telephone interviews alongside face‐to‐face and virtual video options, and as a result, specialised equipment was purchased to facilitate these interviews.

**Figure 2:**
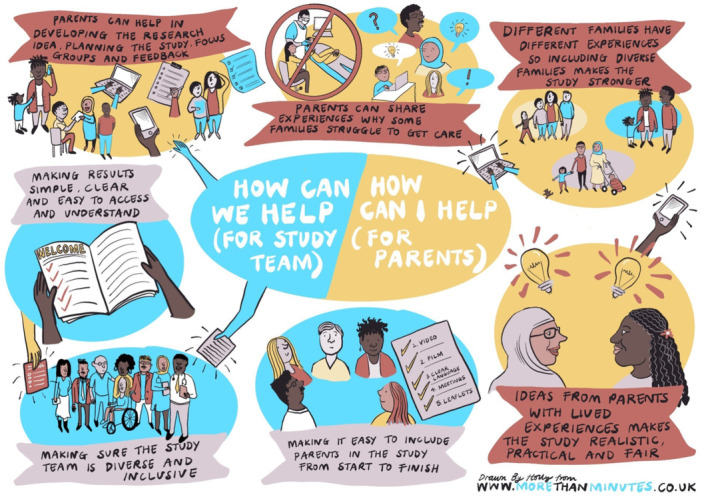
Patient and Public Involvement summary.

PPIE activities led to both short‐ and long‐term benefits. Short‐term benefits included improved accessibility (especially for underserved groups) through simplified study documents, a visual topic guide, flexible interview scheduling, and the option of telephone interviews. Long‐term benefits included the identification of relevant themes, increased awareness of communication difficulties, improved engagement with underserved groups, and a strong foundation for co‐produced research in the future. Parents suggested accessible ways to disseminate findings, including a visual summary of PPIE (Figure 2) and an animation video. The PPIE discussions prompted changes within CLAPA to better acknowledge diverse perspectives. The visual topic guide, visual summary and animation storyboard were particularly well‐received and will be used in future research projects.

HIAT informed consideration of equity, GRIPP2 guided reporting and transparency, and PiiAF was used to assess impact. PPIE activities were grouped according to these domains (equity, transparency and impact), which informed the analytical table (Table 1). This approach ensured that PPIE activities were not only described but also examined in terms of equity, transparency, and impact, improving the relevance, accessibility, and overall quality of the HANDCLAP study.

**Table 1:** PPIE analytical domains.

Activity	Input from parents	Impact on the study	Framework	Analytical domain
PPIE group discussion	Highlighted communication and social factors (language, education, access)	Refined study focus and key themes	HIAT, PiiAF	Equity, Impact
Study documents	Documents difficult to understand	Simplified materials for better accessibility and readability	GRIPP2, HIAT	Transparency, Equity
Vignette	Shared different lived experiences	Informed visual topic guide (Figure 1)	HIAT	Equity
Interview guide	Suggested culturally relevant questions	Revised topic guide to reflect experiences	HIAT	Equity
Interview mode	Recommended telephone interviews	Introduced telephone option	PiiAF, HIAT	Impact, Equity
Recruitment	Highlighted challenges for some groups	Adapted strategy to include underserved groups	HIAT	Equity
Visual tools	Suggested use of visual aids	Developed visual guide (Figure 1)	GRIPP2, HIAT	Transparency, Equity
Analysis	Described overlapping barriers	Included intersecting barriers in analysis	HIAT, PiiAF	Equity, Impact
Dissemination	Suggested accessible outputs	Developed visual summary and animation (Figure 2)	GRIPP2, HIAT, PiiAF	Transparency, Equity, Impact

### 3.5 Discussion

The population of the UK is diverse, with people from a range of cultural and socioeconomic backgrounds. An inclusive and flexible study design is therefore essential, as achieving such diversity in real‐life research is challenging.

PPIE in research is not just a tick‐box exercise but can reveal perspectives that may not be immediately apparent to researchers and clinicians. At the outset of this study, we assumed that limited availability of dentists would be the main barrier to accessing general dental care. However, parents identified communication as a key barrier, including challenges between primary, secondary, and community care, as well as individual communication difficulties.

Parents also highlighted additional factors such as lack of trust in general dentists' and social determinants, including education. These insights were possible through engagement with a diverse group and highlights the importance of including a wide range of lived experience in research. The HIAT further emphasises how research may unintentionally create inequalities, if involvement is limited to less diverse groups.

PPIE in the project began at the proposal stage, contributing to the development of a more relevant and accessible study. Input from parents improved the clarity and readability of research documents and ensured that the topic guide reflected real‐life experiences related to health inequalities. Early parents' involvement also supported the inclusion of culturally sensitive approaches and strengthened the relevance of the research to all stakeholders.

However, achieving diversity within the PPIE group was not straightforward. The response to the national advertisement by CLAPA was less diverse than expected. Greater diversity was achieved through targeted local engagement and by using clear, accessible communication, which avoided assumptions. Collaboration with the CLAPA further enhanced cultural sensitivity and contributed to organisational changes that supported more inclusive involvement. These findings highlight that inclusive PPIE requires active effort rather than reliance on passive recruitment strategies.

Planning and delivering PPIE requires time and resources. Some parents in the PPIE group (n = 2) did not respond despite receiving two reminders, which may reflect competing priorities or limited engagement. Although some groups are described as hard to reach, this study found that meaningful engagement is possible with additional time and effort. The diversity within the research team also supported a more practical understanding of cultural differences and helped avoiding exclusion of participants.

Although the evidence base of PPIE has expanded significantly over the past decade, reporting has been inconsistent and incomplete. Insufficient information is available regarding the context, process, and impact of public involvement, and conceptual or theoretical reporting is rare [7]. It is difficult for an early‐career researcher to appreciate the structural determinants of inequalities, implicit bias, the impact of PPIE, and how a study can cause inequalities. The HIAT, GRIPP2, and PiiAF frameworks supported a more structured and transparent approach to PPIE. While there is some overlap between these frameworks, each contributed to a more comprehensive understanding of PPIE. Together, they supported a more deliberate and reflective approach to embedding equity, transparency, and impact within the study.

PPIE influenced the HANDCLAP study beyond process improvements by shaping the focus of the qualitative research. This ensured that the study explored issues relevant to families rather than relying only on researcher and clinician assumptions. While PPIE did not directly generate findings of the qualitative study, it influenced what was explored and how, helping to make the findings more meaningful and applicable. These PPIE approaches can be adapted to other health research settings by focussing on the principles rather than the specific methods. Involving diverse contributors, using structured frameworks, and maintaining engagement throughout the research process were key in this study and are relevant in other contexts. However, they need to be adapted to the population and setting, for example in how participants are recruited, how discussions are conducted, and how information is shared.

### 3.6 Dissemination

PPIE participants reviewed the visual topic guide, which was circulated to the parents via email. They wished to be informed about the results of this study in plain language and to be involved in future studies relating to cleft dental care. The researchers planned to disseminate the study through meetings, conferences, webinars, and publications to key stakeholders, including dentists, nurses, physicians and cleft lip and palate specialists.

Dissemination to parents is planned via an animated video through the charity and Hospital websites. Future plans include review of animation on the charity website by a diverse group of parents, identifying opportunities for wider dissemination and feedback for further refinements. Additionally, links to all peer‐reviewed journal publications with plain‐language summaries related to this research, will be posted on the CLAPA website. A visual summary clarifying the benefits of PPIE to the research team and to parents is presented in Figure 2. The dissemination of verbal information to parents in clinics would be important to avoid excluding parents without access to technology.

### 3.7 Critical Reflections

The proposal development stage of the project was not co‐developed; however, feedback was sought from a lay person after the proposal had been drafted. PPIE input was then incorporated throughout the project including dissemination. The PPIE experience provided a strong foundation for developing a co‐produced project in the future. PPIE discussions developed a focus on intersectionality, as its significance was appreciated during group discussions. Recruiting members of a PPIE group from diverse communities was challenging, as those who typically contribute to PPIE do not reflect the diversity of the population. The PPIE group included parents from the Cheshire, Merseyside, and North Wales regions (local to the research team), as well as nationally in the UK through CLAPA. Including parents whose first language was not English provided a different perspective during PPIE discussions, which may otherwise have been overlooked. This was reflected in the themes and topics for qualitative study developed after PPIE discussion. Participation in the NIHR ARC NW Internship provided the guidance needed by the principal investigator (early career researcher) to apply HIAT, GRIPP2, and PiiAF frameworks and develop a reflective approach to PPIE.

### 3.8 Conclusion

Using frameworks such as HIAT, GRIPP2, and PiiAF in health inequalities studies improves the fairness and applicability of research. Without PPIE, researchers may miss important insights into social and communicative aspects of research. PPIE requires additional funding, time, and effort, but ensures equity and practicality in research. Ensuring diversity in research is difficult; however, it makes the results more generalisable.

## Author Contributions

**Madhavi Seshu:** conceptualization, funding acquisition, writing – original draft, methodology, resources, project administration, visualization, investigation, formal analysis, data curation, writing – review and editing. **Judith Humphreys:** writing – review and editing, validation, formal analysis. **Gillian McCarthy:** writing – review and editing, conceptualization, methodology, validation. **Alison Mcloughlin:** supervision, writing – review and editing. **Sondos Albadri:** conceptualization, methodology, validation, formal analysis, supervision, funding acquisition, visualization, writing – review and editing.

## Disclosure

The views expressed in this publication are those of the authors and not necessarily those of the National Institute of Health and Care Research, the National Health Service, or the Department of Health and Social Care.

## Ethics Statement

IRAS ID 330971/Wales REC4, Wrexham, 24 January 2024. Written informed consent was obtained from participants in the PPI discussion group.

## Conflicts of Interest

The authors declare no conflicts of interest.

## Data Availability

The data that support the findings of this study are available on request from the corresponding author. The data are not publicly available due to privacy or ethical restrictions.
